# Evaluation of the Morphometry of Sperm from the Epididymides of Dogs Using Different Staining Methods

**DOI:** 10.3390/ani11010227

**Published:** 2021-01-18

**Authors:** Anna Wysokińska, Ewa Wójcik, Angelika Chłopik

**Affiliations:** Faculty of Agrobioengineering and Animal Husbandry, Siedlce University of Natural Sciences and Humanities, 08110 Siedlce, Poland; ewa.wojcik@uph.edu.pl (E.W.); angelikach13@wp.pl (A.C.)

**Keywords:** canine spermatozoa, epididymis, sperm morphometry, staining methods

## Abstract

**Simple Summary:**

Sperm morphometry is an important indicator of the quality of the spermatozoon and its effectiveness in fertilizing the egg. The morphological analysis of sperm collected from dog epididymis may be helpful in the diagnosis of reproductive disorders, valuable for breeding. There are no studies evaluating the morphometry of sperm in individual parts of the epididymal duct in dogs. For this reason, a study was carried out to evaluate the morphometry of sperm from the epididymides of dogs (caput, corpus, cauda) subjected to routine castration. This assessment was performed with the use of four staining methods (DiffQuick (DQ), SpermBlue (SB), eosin-nigrosin (EN) and eosin-gentian (EG)), popular in the assessment of sperm morphology and morphometry. The morphometric dimensions of sperm vary according to the segment of the epididymal duct, from which the cells are collected. Sperm taken from cauda of the epididymides had the smallest dimensions. These were sperm cells with smaller head dimensions (length, area, perimeter) and shorter length of the tail and the sperm total length than the sperm collected from corpus and caput. The study is especially important because it provides a more complete picture of the morphometry of sperm collected from the epididymides of dogs. Knowledge of the sperm morphometric dimensions can be helpful in developing semen preservation methods and assisted reproduction techniques. The combined use of multiple sperm staining techniques enables a more precise evaluation of male reproductive cells. These methods are appropriate for the evaluation of sperm structure, and the possibility of using all four methods enables a full characterization of sperm collected from the caput, corpus and cauda of the epididymides of dogs.

**Abstract:**

Evaluation of sperm morphometry is an important criterion in the diagnosis of a male animal’s suitability for breeding. The aim of the study was to evaluate the morphometry of sperm from the epididymides of dogs subjected to routine castration using various staining methods. The study was carried out on semen collected from ten healthy dogs. Gonads were obtained from each dog during routine castration at a veterinary surgery. Then, the epididymides (caput, corpus, cauda) were isolated from the gonads, semen was collected from them and microscope slides were prepared. The slides for evaluation of sperm morphometry were prepared by four methods: DiffQuik, SpermBlue, eosin-nigrosin and eosin-gentian. A total of 2400 sperm were analyzed (240 sperm from the dog). The sperm collected from the caput and corpus of the epididymis were found to have larger heads and tails than those collected from the cauda of the epididymis. The staining method was shown to affect the morphometry of sperm taken from the epididymides of dogs. The staining methods differentiate the dimensions of the head of sperm in different parts of the epididymis but do not affect the length of the sperm tail. The occurrence of differences in the head dimensions of sperm may be linked to the use of different fixatives and chemical reagents in the staining procedure. Sperm stained by the EN method had the smallest head and tail dimensions. The greatest head area was noted in the sperm stained by the EG method. In the slides stained by the SB method, the sperm heads were relatively long but narrow. The methods used are suitable for the evaluation of sperm structure, and the possibility of using all four methods enables a full characterization of sperm collected from the caput, corpus and cauda of the epididymides of dogs.

## 1. Introduction

The spermatozoon is a male reproductive cell with a very complex morphological structure, which for many years has remained in the broad range of interest of numerous researchers. Dog spermatozoa often have specific traits that distinguish them from the sperm of other mammalian species. The specific maturation process of sperm, accompanied by changes in their structure, begins to take place during the passage of semen through the epididymal duct. Evaluation of the morphometric structure of spermatozoa can be an indicator of their fertilization capacity. Studies on sperm morphometry in various mammalian species [1,2,3,4] have shown a link between sperm dimensions and fertility. Sperm morphometry is evaluated using traditional microscopy with image analysis software, as well as increasingly popular Computer Assisted Semen Analysis (CASA) [5,6], which are believed to ensure more reliable and repeatable results [7,8]. However, irrespective of the method used to evaluate sperm morphometry, it is important to take into account the effect of various factors that may influence variability in the results [9]. Some authors have indicated that slide preparation and staining methods can significantly affect the morphometric dimensions of sperm [10,11]. This effect is due to the means of slide preparation, the type of staining reagents, the temperature, and the type and quality of equipment [12].

Most studies concern semen collected as a result of ejaculation. There is little information about the morphometry of sperm collected from the epididymides. In view of the increasingly common reproductive disorders in valuable male dogs used for breeding, there is a need to look for other methods of obtaining semen. An alternative may be the acquisition of sperm from the epididymides [13]. Therefore, learning the structure of sperm located in the epididymal duct can be helpful in developing better and more effective semen preservation methods and assisted reproduction techniques. Another argument in support of research on semen collected from the epididymis is the increasingly common occurrence of various diseases that may eliminate genetically valuable individuals from breeding. Thus there is a need to evaluate the degree of development of sperm collected from different segments of the epididymides of dogs.

The aim of the study was to evaluate the morphometry of sperm from the different parts of epididymides of dogs (caput, corpus, cauda) subjected to routine castration. This assessment was performed with the use of four staining methods.

## 2. Materials and Methods 

The study was carried out in strict compliance with the recommendations in Directive 63/2010/EU and the Journal of Laws of the Republic of Poland of 2015 on the protection of animals used for scientific or educational purposes. The study was approved by the Polish Local Ethics Committee, Warsaw, Poland (Number: 51/2015) and by the Polish Laboratory Animal Science Association (Numbers 3235/2015; 4466/2017).

### 2.1. Animals and Semen Collection

The study was conducted on sperm collected from ten healthy dogs of different breeds, aged from 4 to 9 years. Gonads were obtained from each dog during routine castration at a veterinary surgery. The testicles together with the epididymides were placed in sterile plastic containers in 0.9% NaCl solution at 37 °C and transported within 2 h to the laboratory for analysis. The gonads were washed with PBS (Adlab, Lublin, Poland), and the epididymides were isolated. Dissection was performed carefully to avoid sectioning of blood vessels. After isolation, the epididymides were divided into segments using surgical forceps. The parts of the epididymis (caput, corpus and cauda) were identified according to Trasler et al. [14] and Kunkitti et al. [15]. Samples obtained from the epididymides (right and left) of the same animal were pooled. Each part (caput, corpus and cauda) was placed in a separate Petri dish and washed with PBS (Adlab, Lublin, Poland) at 37 °C. Sperm was collected by making a cut in each section of the epididymal duct with a sterile scalpel. The sperm was diluted with about 2–3 mL of 99.5% HEPES solution (titration) (Pol-Aura, Dywity/Olsztyna, Poland).

### 2.2. Staining Methods

Slides for evaluation of sperm morphometry were prepared by four methods: DiffQuick (DQ), SpermBlue (SB), eosin-nigrosin (EN) and eosin-gentian (EG).

#### 2.2.1. DiffQuick Staining

A 5 µL volume of semen was deposited on a microscope slide, and then a smear was prepared and left to dry for 1 h. The smears were stained with DiffQuik (Ral diagnostics, Martillac, France) by immersing them for 25 s in three solutions, one after the other (fixative-methanol, solution I-buffered solution of Eosin Y and solution II-buffered solution of thiazine dyes (cationic dyes) consisting of methylene blue and Azure A). They were then rinsed with distilled water and air-dried [16].

#### 2.2.2. SpermBlue Staining

A 10 µL drop of semen was transferred onto a microscopic slide, smeared, and air-dried at room temperature. After drying, the smear was stained with SpermBlue stain (Microptic SL, Barcelona, Spain) for approx. 20–25 min according to the staining procedure developed by van der Horst and Maree [17]. The stained slides were rinsed with distilled water and air-dried.

#### 2.2.3. Eosin-Nigrosin Staining

A 5 µL volume of semen and 10 µL of stain consisting of 5% Eosin Bluish solution (Carl Roth Gmbh + Co. KG, Karlsruhe, Germany) and 10% nigrosin (Sigma-Aldrich, St. Louis, MO, USA) in 1:4 proportions were applied to a microscope slide heated to about 36 °C. Then a smear was prepared and dried at room temperature.

#### 2.2.4. Eosin-Gentian Staining

A 5 µL volume of semen was applied to a microscope slide heated to about 36 °C and smeared. When the smear had dried, it was fixed for 5 min in 96% ethanol solution, rinsed with water, and stained with suitably prepared stains according to the procedure described by Wysokińska and Kondracki [11].

### 2.3. Morphometric Measurements

A total of 120 microscope slides were prepared (12 slides for each dog: 4 slides per epididymal segment, 1 per staining method). In each slide, measurements were made of 20 well-formed sperm (with no morphological defects in the head or tail and no cytoplasmic droplets). A total of 2400 sperm were analyzed (240 sperm from the dog). The measurements were made with a Nikon E-50i light microscope (Tokyo, Japan) equipped with aDSFi3 digital camera and NIS-Elements D5 software. The microscope image of sperm was obtained at 1000× magnification. There were morphometric evaluations using the graphics tablet Wacom Intuos driver 6.3.17 (Krefeld, Germany) equipped with very sensitive digital pen. The following parameters were included in the sperm measurements: head length, head width, head surface area, head perimeter, tail length and total sperm length. The following head shape parameters were calculated from the sperm head measurements: ellipticity (length/width), elongation (length − width)/(length + width), rugosity (4π × area/perimeter^2^), and regularity (π × length × width/4 × area).

### 2.4. Statistical Analysis

The data were tested for normality using Shapiro–Wilk test. The results are presented as mean ± standard error of the mean (SEM). Analysis of variance of the results was performed using Statistica v13.1 software (StatSoft, Tulsa, OK, USA). The data were statistically analysed using the following mathematical model:

*Y_ijk_* = µ + a_i_ + b_j_ + ab_ij_ + *ε_ijk_* (1)

where *Y_ijk_*—value of the analyzed parameter; µ—populational mean; a_i_—the effect of epididymal segment; b_j_—the effect of staining method; ab_ij_—the effect of interaction between factors; *ε_ijk_* —error.

The significance of differences between groups was determined by the Tukey’s test at *p* < 0.05. Additionally, Pearson’s linear correlation was applied to identify the relation between the sperm morphometry and staining methods. The results were considered significant if *p* < 0.05.

## 3. Results

The mean sperm dimensions and mean head shape indices of sperm from each part of the epididymal duct are presented in Table 1. Analysis of the data revealed differences in the dimensions of the heads and tails of sperm depending on the segment of the epididymides. Sperm collected from the caput and corpus of the epididymides had larger head dimensions than sperm from the cauda of the epididymides. These were sperm with longer heads with a larger surface area and perimeter than sperm obtained from the cauda of the epididymides (*p* < 0.05). Similar differences were observed for tail length and total sperm length. Sperm from the cauda of the epididymis had a mean 1.31 µm and 1.73 µm shorter tails than sperm from the caput and corpus, respectively, and a mean 1.47 µm and 1.94 µm smaller total length (*p* < 0.05). No differences were found for the shape indices of the sperm head between segments of the epididymis.

Table 2 presents data characterizing the dimensions and head shape indices of sperm depending on the staining method used. The data show that the staining method influences the morphometry of sperm collected from the epididymides of dogs. Sperm stained by the EN method had the smallest head and tail dimensions. They had shorter heads with a smaller surface area and perimeter than sperm stained by the EG, DQ and SB methods (*p* < 0.05). Sperm stained by the EN method also had significantly shorter tails and a smaller total length than sperm stained by other techniques (*p* < 0.05). The greatest head area was noted in the sperm stained by the EG method. These sperm had the longest tails and the greatest total length in comparison to the other staining methods. Sperm stained by the DQ method had longer heads than sperm stained by the EG and EN methods (*p* < 0.05). In the slides stained by the SB method, the sperm heads were relatively long but narrow. The ellipticity and elongation indices were highest for the heads of sperm stained by this method.

**Figure 1:**
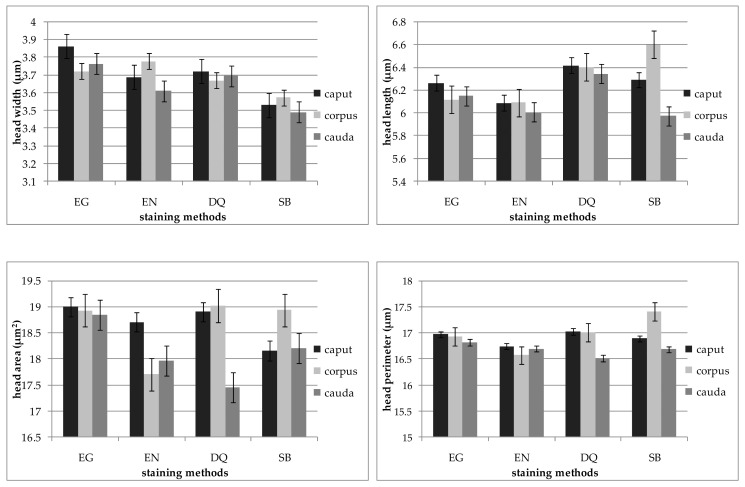
Morphometric dimensions of the heads of sperm collected from the caput, corpus and cauda of the epididymides depending on the staining method (EG, EN, DG, SB). Bars represent means ± SEM (number of analyzed cells = 2400).

Sperm taken from the cauda of the epididymides stained with EN and SB methods had shorter and narrower heads than sperm from the corpus and caput (Figure 1). The longest sperm heads stained by the SB method were obtained from the corpus of the epididymides, as shown in Figure 1. Moreover, in the slides stained with SB, sperm collected from the corpus of the epididymis had heads with a large perimeter. For these morphometric measurements of sperm obtained from the corpus of the epididymides, significant correlations were found between the SB and DQ staining methods (Table 3). Correlations between the DQ staining methods and other methods (EG, EN, SB) were shown for the length of the sperm head collected from the caput and corpus of the epididymis. Positive and high correlation coefficients were also found for the length of the sperm head collected from the cauda of the epididymides between the DQ methods and EG and EN staining (*r* = 0.71 and 0.57).

**Figure 2:**
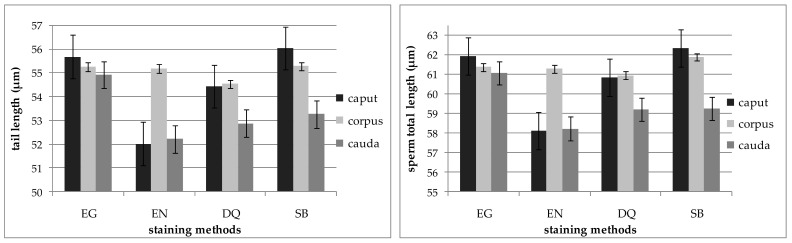
Tail length and total length of sperm collected from the caput, corpus and cauda of the epididymides depending on the staining method (EG, EN, DG, SB). Bars represent means ± SEM (number of analyzed cells = 2400).

In semen collected from the cauda of the epididymides, shorter tails and shorter total sperm length were observed in comparison to semen collected from the caput and corpus of the epididymides (Figure 2).

## 4. Discussion

Male reproductive cells undergo many transformations, first during spermatogenesis in the testes, and later during maturation in the epididymides. These changes affect both the head and tail of the sperm. In our study, well-formed sperm cells were included in the evaluation of sperm morphometry. Therefore, it can be assumed that not all sperm is transformed while passing through the epididymal duct. The analysis of sperm morphometric measurements made in our study indicates the existence of differences in the size of sperm heads in individual segments of the epididymis. The results of our study showed that sperm collected from the cauda of the epididymides had smaller head dimensions than sperm collected from the caput and corpus of the epididymides. Research on the semen of stallions has shown that sperm with smaller heads are more effective in fertilizing the egg [1]. Thus it is likely that the dimensions of the sperm head may be an indicator of the fertilization capacity of the male reproductive cell. The morphometric parameters of the sperm head may reflect the structure of chromatin, which fills the nucleus and occupies most of the sperm head [18]. The size of the sperm head depends on the degree of chromatin condensation. Chromatin condensation within the nucleus of the sperm head begins in the male gonad and continues as the semen moves through successive sections of the epididymis, with the sperm head decreasing in size [19]. The specific nature of chromatin packing in the sperm cell nucleus is associated with protamination, involving the replacement of histones with protamines [20]. Histones are twice the size of protamines, so the exchange of these proteins causes the area occupied by chromatin in the sperm head nucleus to decrease. Therefore any abnormalities in the structure and shape of the spermatozoon may be linked to disturbed DNA structure [21]. This in turn is linked to the presence of histone residues, due to disturbances in the protamination process. Such sperm are characterized by chromatin instability. A relationship has been shown between the morphometry of the sperm head and the sperm DNA fragmentation index [22]. Urbano et al. [22] analyzed four subpopulations of sperm and found that the heads of sperm with small dimensions and a rounded shape had a higher DNA fragmentation index. This suggests that the shape and size of the sperm head are important criteria in classifying male gametes in terms of their fertilization capacity. This is particularly important in the case of sperm collected from the epididymides. The present study showed that the head dimensions of sperm collected from the caput and corpus of the epididymis are similar, but sperm from the cauda of the epididymis have smaller heads. Our study also found that sperm from the cauda of the epididymis had shorter tails than sperm from its corpus and caput.

In a study conducted on sperm collected from feline epididymides, smaller dimensions of sperm heads in the epididymal cauda were found than in the caput and corpus [23]. The study of these authors also found that the sperm head dimension was correlated with chromatin decondensation, and the sperm head size decreased as the sperm were transported through the three epididymal regions. Most studies on sperm morphometry rely on measurements of sperm heads, often using the CASA system [24,25]. These measurements are more objective than manual ones because a large number of cells can be measured, but in most cases, they do not include measurements of the sperm tail. The sperm tail is an important structure that is primarily responsible for the motility of the sperm. In our study, the tail dimensions of sperm were smaller in the cauda of the epididymis than in its caput and corpus. Sperm collected from this part of the epididymis also had the smallest dimensions for the entire sperm. Research on semen collected from the epididymides of dogs has shown that as the sperm pass through the caput, corpus and cauda of the epididymides, the number of cytoplasmic droplets decreases, especially proximal droplets, and thus the cells acquire the capacity for movement [26]. Semen from the cauda of the epididymides has proved resistant to superoxide anion damage, which is correlated with sperm motility [27]. Thus, it can be concluded that the dimensions of the sperm tails determine variation in the activity of sperm. The shorter ones from the cauda of the epididymides in our study as compared to the caput and corpus may have better motility. Opinions on the influence of the tail length of sperm on their fertilization capacity are divided. Some believe that sperm with longer tails have better motility, but the motility of these sperm does not last as long as in sperm with shorter tails [28]. However, there is no definitive opinion on semen collected from the epididymides of dogs. Our study demonstrates that sperm isolated from the cauda of the epididymis have shorter tails.

The present study showed that the dimensions of sperm collected from the epididymides of dogs are affected by the staining method. We used four staining methods that are common in evaluations of the morphology and morphometry of human and animal sperm. The eosin-nigrosin (EN) method has been recommended by the WHO for analysis of human sperm but has also found application in the evaluation of the sperm morphology of animals [29]. This method, apart from semen diagnostics in terms of sperm morphology, also enables evaluation of the integrity of the cell membranes of sperm, identifying them as live (unstained heads) and dead (heads stained pink) [30]. The eosin-gentian staining method (EG) is often used to evaluate the morphology and morphometry of farm animals, such as boars [31], bulls [32] or stallions [33]. This method distinctly stains the sperm head and tail, but some authors suggest that it can cause the heads to swell, which has been observed in the case of bird sperm [34]. Another method used to stain the semen of various animal species is DiffQuik [16]. This method is also recommended for evaluation of the morphology of human semen. It is a simple and fast procedure for staining slides [35]. In recent years, the SpermBlue (SB) method has increasingly been used to stain slides. Research on human semen has shown that the SpermBlue method clearly differentiates the individual parts of the spermatozoon and enables precise analysis of each of its structures [17]. The use of an appropriate staining technique is of great importance for correct semen diagnostics. The staining method should interfere as little as possible in the cell structures of the sperm, while at the same time staining the individual elements so that their structure can be precisely evaluated [36]. However, the sperm of different species may react differently to the same reagents used to prepare the slide [17]. Most studies on the effect of the staining technique on the morphology and morphometry of sperm of different animal species have been conducted on ejaculate semen [16,31,32]. There are no clear suggestions regarding staining methods for semen collected from the epididymides of dogs. Our study found that the staining methods cause differences in the dimensions of sperm from different sections of the epididymis, especially in sperm head dimensions. Sperm from the cauda of the epididymides generally had smaller head dimensions than sperm from the caput and corpus of the epididymides. Thus, it is likely that the staining techniques used in the present study can be used to evaluate the morphometry of sperm collected from the epididymides of dogs. Of the four staining techniques used, the smallest sperm dimensions were obtained in the EN method, as shown in Table 2. Similar observations have been made in studies on boar semen, which showed that the EN staining method results in smaller morphometric sperm dimensions than the EG method [31]. Another study showed that the SB staining method has the least effect on dimensions of sperm heads, but this method does not enable a precise determination of the extent of the acrosome [10]. Of the three Hemacolor staining methods, used to assess sperm morphometry, Soler et al. [37] found that the DiffQuik method was the best. Apart from the morphometric dimensions of the sperm head and tail, head shape indices are also important. These indices are most often defined as ellipticity, elongation, rugosity and regularity. In our study, we found differences in the head shape indices of sperm between staining techniques. Sperm stained with the SB and DQ methods had greater ellipticity and elongation than sperm stained by the EG and EN methods. According to Malo et al. [38], sperm with more elongated heads are faster than those with a rounded head shape.

## 5. Conclusions

To sum up, during the passage of semen through successive segments of the epididymides of dogs, changes take place in the morphometric dimensions of the sperm. Sperm collected from the cauda of the epididymides have smaller head and tail dimensions than sperm from the caput and corpus of the epididymides. The staining methods differentiate the dimensions of the sperm heads in different parts of the epididymides but do not affect the length of the sperm tails. Studies carried out on sperm from the epididymides are especially important because they create a number of possibilities in terms of artificial insemination techniques and other methods of assisted reproduction, as well as protection of endangered animal species, including wild carnivores of the family Canidae. Our study contributes new knowledge regarding the changes taking place in sperm structures during their passage through each segment of the epididymides in dogs. The combined use of the staining techniques presented in this study enables a more precise evaluation of male reproductive cells. The findings and their implications should be discussed in the broadest context possible. Future research directions may also be highlighted.

## Figures and Tables

**Table 1:** Sperm morphometry in each segment of the epididymides of dogs (mean ± SEM).

Item.	Epididymal Segment			*p*-Value
	Caput	Corpus	Cauda	
Number of Analyzed Cells	800	800	800	
Head				
Length (µm)	6.28 ± 0.05 ^a^	6.32 ± 0.05 ^a^	6.11 ± 0.05 ^b^	0.01
Width (µm)	3.70 ± 0.03 ^a^	3.67 ± 0.02 ^a^	3.63 ± 0.02 ^a^	0.56
Area (µm^2^)	18.67 ± 0.18 ^a^	18.69 ± 0.13 ^a^	18.12 ± 0.13 ^b^	0.02
Perimeter (µm)	16.90 ± 0.08 ^ab^	17.01 ± 0.08 ^a^	16.70 ± 0.07 ^b^	0.04
**Tail**				
Length (µm)	54.62 ± 0.34 ^a^	55.04 ± 0.35 ^a^	53.31 ± 0.30 ^b^	0.01
Sperm total length (µm)	60.89 ± 0.35 ^a^	61.36 ± 0.35 ^a^	59.42 ± 0.31 ^b^	0.00
**Shape indices**				
Ellipticity	1.72 ± 0.02 ^a^	1.73 ± 0.02 ^a^	1.69 ± 0.02 ^a^	0.44
Elongation	0.26 ± 0.01 ^a^	0.26 ± 0.00 ^a^	0.25 ± 0.00 ^a^	0.33
Rugosity	0.82 ± 0.01 ^a^	0.81 ± 0.00 ^a^	0.82 ± 0.00 ^a^	0.46
Regularity	0.99 ± 0.01 ^a^	0.98 ± 0.01 ^a^	0.96 ± 0.01 ^a^	0.68

^a,b^ Different letters in rows indicato differences (*p* < 0.05).

**Table 2:** Morphometry of sperm collected from the epididymides of dogs depending on the staining method (EG, EN, DQ, SB) (mean ± SEM).

Item	Staining Method				*p*-Value
	EG	EN	DQ	SB	
Number of Analyzed Cells	600	600	600	600	
Head					
Length (µm)	6.17 ± 0.06 ^a^	6.06 ± 0.06 ^a^	6.39 ± 0.06 ^b^	6.27 ± 0.06 ^b^	0.00
Width (µm)	3.78 ± 0.03 ^a^	3.69 ± 0.03 ^b^	3.70 ± 0.03 ^ab^	3.53 ± 0.02 ^c^	0.00
Area (µm^2^)	18.92 ± 0.18 ^a^	18.11 ± 0.18 ^b^	18.46 ± 0.17 ^ab^	18.42 ± 0.15 ^ab^	0.02
Perimeter (µm)	16.93 ± 0.10 ^a^	16.67 ± 0.09 ^a^	16.85 ± 0.09 ^a^	16.97 ± 0.08 ^a^	0.11
**Tail**					
Length (µm)	55.25 ± 0.27 ^a^	53.06 ± 0.53 ^b^	53.94 ± 0.32 ^b^	54.75 ± 0.37 ^a^	0.00
Sperm total length (µm)	61.43 ± 0.28 ^a^	59.12 ± 0.53 ^b^	60.33 ± 0.34 ^ab^	61.02 ± 0.38 ^a^	0.00
**Shape indices**					
Ellipticity	1.64 ± 0.02 ^a^	1.66 ± 0.02 ^a^	1.74 ± 0.02 ^b^	1.79 ± 0.02 ^b^	0.00
Elongation	0.24 ± 0.01 ^a^	0.24 ± 0.01 ^a^	0.27 ± 0.01 ^b^	0.28 ± 0.01 ^b^	0.00
Rugosity	0.83 ± 0.00 ^a^	0.82 ± 0.01 ^ab^	0.82 ± 0.00 ^ab^	0.80 ± 0.01 ^b^	0.00
Regularity	0.97 ± 0.01 ^a^	0.97 ± 0.01 ^a^	1.01 ± 0.01 ^b^	0.95 ± 0.01 ^a^	0.00

^a,b,c^ Different letters in rows indicato differences (*p* < 0.05).

**Table 3:** Pearson correlation coefficients between the morphometry of sperm collected from the caput, corpus and cauda of the epididymis of dogs and the staining method (DQ—DiffQuik, EG—eosin-gentian, EN—eosin-nigrosin and SB—SpermBlue) (* *p* < 0.05).

Item		Epididymal Segment								
		Caput			Corpus			Cauda		
		DQ	EG	EN	DQ	EG	EN	DQ	EG	EN
Head										
Length (µm)	EG	0.56 *	-	0.30	0.41 *	-	0.45 *	0.71 *	-	0.72 *
	EN	0.33 *	0.30	-	0.62 *	0.45 *	-	0.57 *	0.72 *	-
	SB	0.31 *	0.31 *	0.02	0.46 *	0.22	0.29	−0.16	−0.10	−0.24
Width (µm)	EG	0.24	-	−0.21	0.06	-	−0.13	−0.12	-	−0.07
	EN	0.21	−0.21	-	−0.03	−0.13	-	−0.01	−0.07	-
	SB	−0.01	−0.09	0.11	−0.30	−0.11	0.10	−0.03	0.01	−0.07
Area (µm^2^)	EG	0.44 *	-	−0.02	0.16	-	0.07	−0.02	-	0.01
	EN	−0.03	−0.02	-	0.05	0.07	-	0.02	0.01	-
	SB	0.07	0.23	−0.09	0.27	0.30	0.04	0.01	0.21	0.13
Perimeter (µm)	EG	0.41 *	-	-0.03	−0.09	-	0.08	0.29	-	0.18
	EN	0.04	−0.03	-	0.26	0.08	-	0.24	0.18	-
	SB	0.18	0.35 *	0.08	0.34 *	0.28	0.01	−0.09	0.15	0.29
**Tail**										
Length (µm)	EG	−0.24	-	−0.41 *	0.39 *	-	0.27	0.26	-	0.06
	EN	0.30	−0.41 *	-	0.06	0.27	-	−0.12	0.06	-
	SB	0.18	−0.25	−0.01	0.23	0.28	0.34 *	0.05	0.24	0.07
Sperm total length (µm)	EG	−0.09	-	−0.28	0.45 *	-	0.21	0.32 *	-	0.10
	EN	0.38 *	−0.28	-	0.06	0.21	-	−0.06	0.10	-
	SB	0.19	−0.27	0.02	0.24	0.23	0.40*	0.06	0.30	0.11

Where - stands for not determined.

## Data Availability

No new data were created or analyzed in this study. Data sharing is not applicable to this article.
